# Safety of allogeneic bone marrow derived mesenchymal stromal cell therapy in renal transplant recipients: the neptune study

**DOI:** 10.1186/s12967-015-0700-0

**Published:** 2015-11-04

**Authors:** Marlies E. J. Reinders, Geertje J. Dreyer, Jonna R. Bank, Helene Roelofs, Sebastiaan Heidt, Dave L. Roelen, Maarten L. Zandvliet, Volkert A. L. Huurman, Wim E. Fibbe, Cees van Kooten, Frans H. J. Claas, Ton J. Rabelink, Johan W. de Fijter

**Affiliations:** Department of Nephrology, Leiden University Medical Center, Albinusdreef 2, 2300 RC Leiden, The Netherlands; Department of Immuno-Haematology and Blood Transfusion, Leiden University Medical Center, Albinusdreef 2, 2300 RC Leiden, The Netherlands; Department of Clinical Parmacy and Toxicology, Leiden University Medical Center, Albinusdreef 2, 2300 RC Leiden, The Netherlands; Department of Surgery, Leiden University Medical Center, Albinusdreef 2, 2300 RC Leiden, The Netherlands; Einthoven Laboratory for Experimental Vascular Medicine, Leiden University Medical Center, Albinusdreef 2, 2300 RC Leiden, The Netherlands

**Keywords:** Allogeneic mesenchymal stromal cells, Renal transplantation, Immune response, Rejection

## Abstract

**Background:**

Mesenchymal stromal cells (MSC) may serve as an attractive therapy in renal transplantation due to their immunosuppressive and reparative properties. While most studies have used autologous MSCs, allogeneic MSCs offer the advantage of immediate availability for clinical use. This is of major importance for indications where instant treatment is needed, for example allograft rejection or calcineurin inhibitor toxicity. Clinical studies using allogeneic MSCs are limited in number. Although these studies showed no adverse reactions, allogeneic MSCs could possibly elicit an anti-donor immune response, which may increase the incidence of rejection and impact the allograft survival in the long term. These safety issues should be addressed before further studies are planned with allogeneic MSCs in the solid organ transplant setting.

**Methods/design:**

10 renal allograft recipients, 18–75 years old, will be included in this clinical phase Ib, open label, single center study. Patients will receive two doses of 1.5 × 10^6^ per/kg body weight allogeneic bone marrow derived MSCs intravenously, at 25 and 26 weeks after transplantation, when immune suppression levels are reduced. The primary end point of this study is safety by assessing biopsy proven acute rejection (BPAR)/graft loss after MSC treatment. Secondary end points, all measured before and after MSC infusions, include: comparison of fibrosis in renal biopsy by quantitative Sirius Red scoring; de novo HLA antibody development and extensive immune monitoring; renal function measured by cGFR and iohexol clearance; CMV and BK infection and other opportunistic infections.

**Discussion:**

This study will provide information on the safety of allogeneic MSC infusion and its effect on the incidence of BPAR/graft loss.

Trial registration: NCT02387151

## Background

Overall kidney graft survival has improved over the past decades, mainly as a result of improvement of first-year graft survival due to better immunosuppressive regimens and overall medical care. However, long-term graft survival remained unaltered over the past two decades mainly because of graft loss due to interstitial fibrosis and tubular atrophy (IF/TA) [1]. The mechanism of IF/TA is thought to be a result of immunologic and non-immunologic causes including calcineurin inhibitor toxicity. More recently, there has been a focus on antibody-mediated rejection indicating an important role for humoral immunity in late kidney allograft failure [2–8].

In order to improve long term graft survival and minimize side effects of the current immune suppressive agents, new therapies are sought. Mesenchymal stromal cell (MSC) therapy constitutes an attractive intervention due to their immunosuppressive and reparative properties and their likely limited side effects [9]. In vitro studies imply that MSCs may play a role in modulation of immune responses. These beneficial immune modulatory effects have been confirmed in experimental models of allo- and autoimmune disorders, including allograft rejection [10–14]. First results of autologous bone marrow (BM) derived MSC therapy after human renal transplantation demonstrate safety and feasibility and illustrate their immune suppressive properties [15–22].

Most studies have used autologous MSCs. However, due to the expansion period, quality controls and logistics, product manufacturing takes several weeks, which is a long period of time for patients in need for acute treatment, for example in calcineurin toxicity and allograft rejection. Allogeneic MSCs offer the advantage of immediate availability “off-the-shelf” for clinical use. Another benefit of using allogeneic MSCs is that the cells can be selectively derived from young donors. This is important because MSC number and functionality has been shown to decrease with age [23–25]. Importantly, a potential disadvantage of allogeneic MSC treatment could be the development of an anti-donor immune response, as has been described in experimental studies [26, 27]. Therefore, it is necessary that we establish whether allogeneic MSC therapy in renal recipients is safe and does not evoke an anti-donor response, which might negatively impact graft function and survival. In the current protocol, allogeneic MSCs are infused at a time point where immune suppression levels are reduced and the graft is at increased risk for developing immune mediated injury. Additionally, a significant proportion of the grafts already has developed signs of fibrosis at this time, a process that might be reduced by the MSCs. Primary endpoints of this study include allograft rejection and graft loss. To minimize the chance of development of anti-donor immune responses and thus allograft rejection our strategy is to select allogeneic MSCs, which have no similarity with the HLA mismatches between the kidney graft and the recipient. A second criteria is that the recipient should have no preformed HLA antibodies directed to the MSCs in order to prevent immune destruction of the MSCs.

## Methods and design

### Objectives and endpoints

The primary endpoint of the current study is safety by assessing biopsy proven acute rejection (BPAR) and graft loss after allogeneic BM derived MSC treatment. The following secondary endpoints will be measured before and after MSC treatment: the level of fibrosis, as determined by quantitative Sirius Red (SR) scoring of renal biopsies; glomerular filtration rate (GFR) calculated by CKD-EPI formula and measured by iohexol clearance; Cytomegalovirus (CMV), BK infection (viremia, disease, syndrome and subtypes of BK viremia) and other opportunistic infections; the presence of anti-human leucocyte antigen (HLA) donor specific antibodies (DSA) and other phenotypical and functional aspects of the donor specific immune response.

### Study design

The current trial is a 12-month, non-randomized, open-label, prospective, single-center study. In total, 10 de novo renal recipients, 18–75 years of age, will be recruited from the transplant clinic of the Leiden University Medical Center (LUMC) and enrolled into the study if they meet the eligibility criteria. Ethical approval was obtained from the Dutch Central Committee on Research Involving Human Subjects (CCMO) and the Dutch competent authority.

### Inclusion criteria

1. Female or male, aged between 18 and 75 years.
2. Subject is willing to participate in the study, must be able to give informed consent and the consent must be obtained prior to any study procedure.
3. Recipients of a first kidney graft from a living-unrelated or non-HLA identical living related donor.
4. Panel Reactive Antibodies (PRA) ≤50 %.
5. Patients must be able to adhere to the study visit schedule and protocol requirements.
6. If female and of child-bearing age, subject must be non-pregnant, non-breastfeeding, and use adequate contraception.

### Exclusion criteria

1. Double organ transplant recipients.
2. BPAR in the 4 weeks before MSC infusion.
3. Patients with evidence of active infection or abscesses (with the exception of an uncomplicated urinary tract infection) before MSC infusion.
4. Patients suffering from hepatic failure.
5. Patients suffering from an active autoimmune disease.
6. A psychiatric, addictive or any disorder that compromises ability to give truly informed consent for participation in this study.
7. Use of any investigational drug after transplantation.
8. Documented HIV infection, active hepatitis B, hepatitis C or tuberculosis according to current transplantation inclusion criteria.
9. Subjects with an active opportunistic infection at the time of MSC infusion [e.g., herpes zoster (shingles), CMV, BK, Pneumocystis jirovecii (PJP), aspergillosis, histoplasmosis or mycobacteria other than tuberculosis].
10. Malignancy (including lymphoproliferative disease) within the past 2–5 years (except for squamous or basal cell carcinoma of the skin that has been treated with no evidence of recurrence) according to current transplantation inclusion criteria.
11. Known recent substance abuse (drug or alcohol).
12. Patients who are recipients of ABO incompatible transplants.
13. Patients with severe total hypercholesterolemia (>7.5 mmol/L) or total hypertriglyceridemia (>5.6 mmol/L) (patients on lipid lowering treatment with controlled hyperlipidemia are acceptable).

All patients will receive alemtuzumab induction therapy at day 0 and day 1 (15 mg subcutaneously) and maintenance triple therapy consisting of prednisone, tacrolimus (Advagraf), and everolimus (Certican). The target of the everolimus is 3–8 ng/mL. The tacrolimus target is 8–10 ng/mL the first 6 weeks after transplantation, 4–7 ng/mL from week 6 to week 24 and will be reduced to targets of 1.5–3 ng/mL after week 24. Patients will receive two doses of a target of 1.5 × 10^6^ selected allogeneic MSCs per/kg body weight at weeks 25 and 26 after transplantation.

### Isolation of bone marrow and MSC production

MSCs for the study will be obtained from an independent third party donor according to the LUMC standardized and approved protocol. All donors will undergo routine examination and screening tests, according to the standard procedures required for BM donors in the LUMC. BM will be aspirated from the posterior iliac crest. A total volume of 100 to 120 mL will be harvested. The processing and expansion of the cells will take place at the Good Manufacturing Practice (GMP) Facility of the LUMC. During the expansion process, population doubling time and cell numbers are monitored as a marker for population fitness and functionality. After approval of in process and final product quality control results, the MSC products will be released for the clinical trial under a EU GMP manufacturers license. Final product release is based on the following criteria: surface marker expression (>90 % of cells CD73^+^/CD90^+^/CD105^+^, ≤1 % of cells CD45^+^ and ≤0.01 % of cells CD3^+^), absence of microbial contamination using culture and mycoplasma PCR, spindle shaped morphology, a colorless cell suspension devoid of cell aggregates, no genetic abnormalities using karyotype analysis and viability.

### Data collection

Patients enrolled in this study will undergo standard pre-transplant work-up, which consists of baseline clinical data (demographics, medical history, current medication, previous blood transfusions, percentage of PRA, infection status, physical examination, laboratory examinations, urinalysis, electrocardiogram and chest X-ray). For women, the menopausal status will be recorded and a pregnancy test will be done prior to transplantation if applicable.

Intraoperative data (warm and cold ischemia time, anatomy of donor and recipient) and background information of the kidney donor (age, gender, race, height, weight, type of allograft (living related or unrelated), infection status, serum creatinine) and HLA (mis)match will also be documented. All immunosuppressive and other drugs used and dosages administered will be recorded during the study.

### Assessment schedule

Subjects will be seen in accordance with the assessment schedule listed in Table 1.

**Table 1:** Assessment schedule

Week	BL	Tx	W24	W25	W26	W27	W28	W30	W34	W38	W42	W46	W52
Informed consent	X												
Medical history	X	X											
Transplantation information		X											
Concomitant medication	X	X	X	X	X	X	X	X	X	X	X	X	X
Physical examination	X	X	X	X	X	X	X	X	X	X	X	X	X
Routine lab	X	X	X	X	X	X	X	X	X	X	X	X	X
Urinalysis	X	X	X	X	X	X	X	X	X	X	X	X	X
CMV, EBV and BK viral load			X	X		X		X		X		X	X
MSC infusion				X	X								
Renal biopsy		X	X										X
Iohexol clearance			X										X
Immune monitoring		X	X	X^a^	X^a^	X			X				X
DSA	X	X	X					X					X
Safety assesment	X	X	X	X	X	X	X	X	X	X	X	X	X

*BL* baseline, *Tx* renal transplantation

^a^before and 4 h after MSC infusion

### Infusion of MSCs

A clinical re-evaluation will be performed before the planned MSC infusion to rule out any contra-indication for administration. A target number of 1,5x10^6^ MSCs per/kg body weight (range 1–2 × 10^6^) will be infused IV within 30 min. Close monitoring of vital signs (temperature, pulse, respiratory rate, blood pressure and oxygen saturation) will be measured before, during and up to 2 h after MSC infusion.

### (Opportunistic) infections

Hepatitis B, C and HIV status will be evaluated routinely within 6 months before transplantation.

CMV (PCR-positive), EBV (PCR-positive), BK-viruria in urine samples and BK-viremia in blood samples (RT-PCR) will be measured as shown in Table 1. In addition subtypes of BK will be determined. Other infections (including urinary tract infections, pulmonary infections, herpes simplex) will be recorded as well. Patients are treated routinely with valganciclovir prophylaxis for 6 months except in case of a CMV negative donor and recipient status. In addition, all patients receive 6 months of cotrimoxazole prophylaxis against PJP.

### Renal function

GFR calculation will be used to determine the renal function [28]. The following abbreviated CKD-EPI formula will be used for GFR estimation: eGFR [mL/min/1.73m2] = 141 × min (SCr/k,1)^α^ × max(SCr/k,1)^−1.209^ × 0.993^age^ × (1.018 if female) × (1.159 if black) (k is 0.7 for females and 0.9 for males, α is −0.329 for females and −0.411 for males). In addition we will measure renal function with iohexol clearance at week 24 and week 52 after transplantation [29].

### Renal biopsy

A standard renal protocol biopsy is performed at transplantation (T = 0) and at 24 weeks after transplantation. At 52 weeks after transplantation a study biopsy is taken to assess the renal histology after MSC infusion. Biopsies are scored according to the Banff criteria and processed for immunohistochemistry (Hematoxylin and eosin staining; staining for CD3, CD4, CD68, FOXp3, C4d and CD20). Tissue will be embedded in paraffin and stained for Sirius Red [30]. The amount of cortical collagen (SR-positive area) will be measured and finally expressed as the percentage of the total analyzed cortical surface.

### Immune monitoring

DSA will be measured by luminex antibody screening and CDC/Flow crossmatch at baseline, before and after MSC infusion, and every time a for-cause allograft biopsy is performed. For immunological monitoring, sera and PBMCs will be collected at various time points post transplantation as described in Table 1. Phenotypical analyses of the different leucocyte subpopulations will be performed similar to our recently described protocol [22] on basis of the immune panels developed and validated for the One Study [31]. These panels identify different subsets of T cells, B cells and DCs. In addition, PBMC proliferation assays will be performed sequentially with the use of frozen PBMCs obtained before transplantation to compare responses to the donor cells of the kidney donor before and after transplantation [32]. PBMCs will be stimulated using CD3/CD28 and analyzed for TH1 (i.e. interleukin-2 and interferon-γ), TH2 (IL-10 and IL-4) and inflammatory cytokines (i.e. tumor necrosis factor-α, TGF-β, IL-1 and IL-6) [33]. The levels of a broad range of systemic pro-inflammatory and anti-inflammatory cytokines and chemokines will be measured by multiplex assays [34].

### Risk–benefit assessment

With the use of allogeneic MSCs, renal recipients who have an acute indication for treatment could benefit from this therapy. A potential danger of allogeneic MSCs could be sensitization of the recipient and an increased risk for allograft rejection. We argue that the absence of significant alloimmune reactions in patients receiving allogeneic MSCs in previous trials [35, 36] and the time of MSC administration beyond 24 weeks after transplantation in combination with accurate immune testing before MSC infusion warrant this study. In addition, specific HLA-mismatch criteria further minimize the risk of sensitization: MSCs will be selected to have no similarity with the HLA mismatches between the kidney graft and the recipient.

## Discussion

In renal transplantation MSC therapy could be attractive in view of its potent effects on immune cells and its reparative properties. The addition of MSCs to the current immune suppressive strategies could help reduce the level of the toxic immune suppressants and limit the fibrosis reaction and immune mediated injury. We envisage that this could be a step forward to prolong allograft survival. In this phase I study we will test whether the administration of selected allogeneic MSCs is safe and does not increase the incidence of BPAR and/or graft loss.

Allogeneic MSCs offer several key advantages over autologous MSCs, including the possibility of donor selection and availability “off-the-shelf” for clinical use without the delay required for expansion. However, a potential danger of allogeneic MSCs could be sensitization with an increased risk for antibody mediated allograft rejection [26, 27]. Therefore endpoints in our study are focused both on allograft rejection and on immune monitoring before and after MSC therapy. Immune monitoring will be performed according to the methods validated in The One study as described by Streitz et al., and as also used in our recent Triton protocol where autologous MSCs are administered early after transplantation [22, 31]. For this immune monitoring strategy, six panels are used to analyze the immune response, including the general immune status, T cell subsets, αβ + T cells and γδ + T cells, T cell activation, T cell memory and regulatory T cells. In addition, B cell and dendritic cell (DC) subsets will be measured. Besides this robust and validated immune monitoring, DSAs and their complement binding capacities will be determined at several time points. Various studies have reported the clinical relevance of the occurrence of anti-HLA antibodies and DSAs in transplantation setting [2–4, 7, 8]. A study performed in 2009 reported a significantly lower 10-year graft survival in patients with DSAs after renal transplantation compared to the DSA negative group (49 vs. 83 %) [5]. More recent studies confirmed the importance of these de novo HLA specific DSA as a major cause of long-term allograft failure. In the majority of the studies, the incidence of de novo DSA is below 15 % [37, 38]. Not all DSAs, however, appear to be equally harmful. Antibodies directed at HLA class II and the capacity to fix complement appear to give worse outcome with more ABMR and higher risks of graft loss [39–42].

The formation of DSAs and the development of rejection after allogeneic MSC infusion has been reported in several animal studies especially with the use of donor derived MSCs [43–45]. In mice receiving an allogeneic BM transplantation the addition of autologous MSCs has been compared to donor derived MSCs. The infusion of donor MSCs was associated with a higher incidence of rejection of the BM transplant [43]. In another study, in renal transplantations in mice, increased allograft rejection was reported and an increase of DSAs was seen when donor derived MSCs were administered 4 days before transplantation [45]. In contrast, the use of third party MSCs caused long term heart graft survival in rats, even when retransplanted in a second host without the use of immune suppression [46].

In the human setting allogeneic MSCs have been proven to be safe in a clinical study for the treatment of acute kidney injury and also clinical studies in haematopoiectic stem cell transplantation have reported no related severe adverse effects [14, 47, 48]. Allogeneic MSCs have also been used in the Poseidon trial where autologous and third party allogeneic MSCs for the treatment of ischemic cardiomyopathy were compared. Transendocardial injection of both autologous and allogeneic MSCs was safe with low rates of related adverse events. Importantly, only 1 patient developed low levels of de novo DSAs after allogeneic MSC injection [35]. Few studies have focused on allogeneic MSCs in transplant recipients. The increase in allograft rejection or DSAs was not reported in a recent pilot study where six renal transplant recipients received donor derived MSCs combined with low dose tacrolimus. The therapy was safe and prevented acute rejection after renal transplantation [36], however the induction of HLA antibodies was not tested in this study.

To minimize the risk of an anti-donor immune response in our study, we have chosen in our protocol to use selected allogeneic MSCs based on the before mentioned HLA criteria. In addition, patients will receive alemtuzumab induction, which according to several studies might reduce the incidence of BPAR. As maintenance therapy a combination with everolimus is used. In a heart transplant model MSC monotherapy inhibited acute graft rejection and the combination of MSCs with rapamycin induced donor-specific allograft tolerance. In the tolerant recipients, MSCs migrated to the transplanted heart and various lymphoid organs. In addition a high frequency of Tol-DCs and CD4^+^CD25^+^Foxp3^+^ T cells was found in these animals [49].

Besides extensive immune monitoring, an important secondary endpoint of our study is renal function before and after MSC treatment. In the study by Perico, renal function declined when the MSCs were administered 7 days after transplantation [16]. The hypothesis was that the subclinical inflammatory environment of the graft post transplantation favoured recruitment and activation of the MSCs, and thereby promoting a pro-inflammatory milieu with eventual acute renal dysfunction. Because the renal dysfunction could depend on the timing of infusion, in their next study MSCs were administered the day before kidney transplantation and in these patients no deterioration of renal function was seen [50]. Renal dysfunction was also not observed in our former study on a patient cohort that was treated more than 6 weeks after transplantation [17].

In addition, fibrosis in the allograft will be measured before and after MSC treatment since this is a hallmark of patients who develop allograft dysfunction [51]. It would be of interest to test whether allogeneic MSCs influence this process and could serve as therapy for IF/TA. Moreover, CMV, BK and opportunistic infections, will be monitored before and after MSC treatment. In our previous small study we found an increased incidence of viral infections [17], however this was not observed in the study by Tan et al. [19].

In this study we use bone marrow derived MSCs. An interesting development for future studies is the great therapeutic potential of allogeneic stromal cells harvested from Wharton’s Jelly (WJ) of the human umbilical cord [52, 53]. In comparison to bone marrow derived MSCs, the isolation efficiency from WJ MSCs is greater while they have also a higher proliferative capacity [54, 55]. These are important properties since large number of cells is needed for MSC treatment. An additional option is to set up a bank of MSC donors, homozygous for the most common HLA haplotypes, which can provide a well matched MSC graft for a significant number of recipients [56].

Taken together, allogeneic MSC may be an important treatment option for recipients of a renal transplant, especially for indications where autologous MSCs cannot be realized logistically. We however believe, that we should accurately study the immune response and incidence of allograft rejection, which could be elicited by allogeneic MSCs, before we move on to larger studies.

## Abbreviations

BL: baseline
BM: bone marrow
BPAR: biopsy proven acute rejection
CCMO: Central Committee on research involving human subjects
CMV: cytomegalovirus
CNI: calcineurin inhibitors
DSA: donor specific antibody
EBV: epstein barr virus
GFR: glomerular filtration rate
GMP: good manufacturing practice
HLA: human leucocyte antigen
IF/TA: interstitial fibrosis and tubular atrophy
LUMC: Leiden University Medical Center
METC: medical ethics committee
MSCs: mesenchymal stromal cells
PBMC: peripheral blood mononuclear cell
PRA: panel reactive antibody
PJP: pneumocystis jiroveci pneumonia
SCr: serum creatinin
SR: sirius red
Tx: renal transplantation
WJ: wharton jelly
